# Supplementary material for: Modelling Virgin Olive Oil Potential Shelf-Life from Antioxidants and Lipid Oxidation Progress
Source: Antioxidants (Basel). 2022 Mar 11;11(3):539. doi: 10.3390/antiox11030539 (PMC8944963; doi:10.3390/antiox11030539)
Supplement: Supplementary file 1 [file antioxidants-11-00539-s001.zip › antioxidants-1620016-supplementary.pdf]

Supplementary Materials:

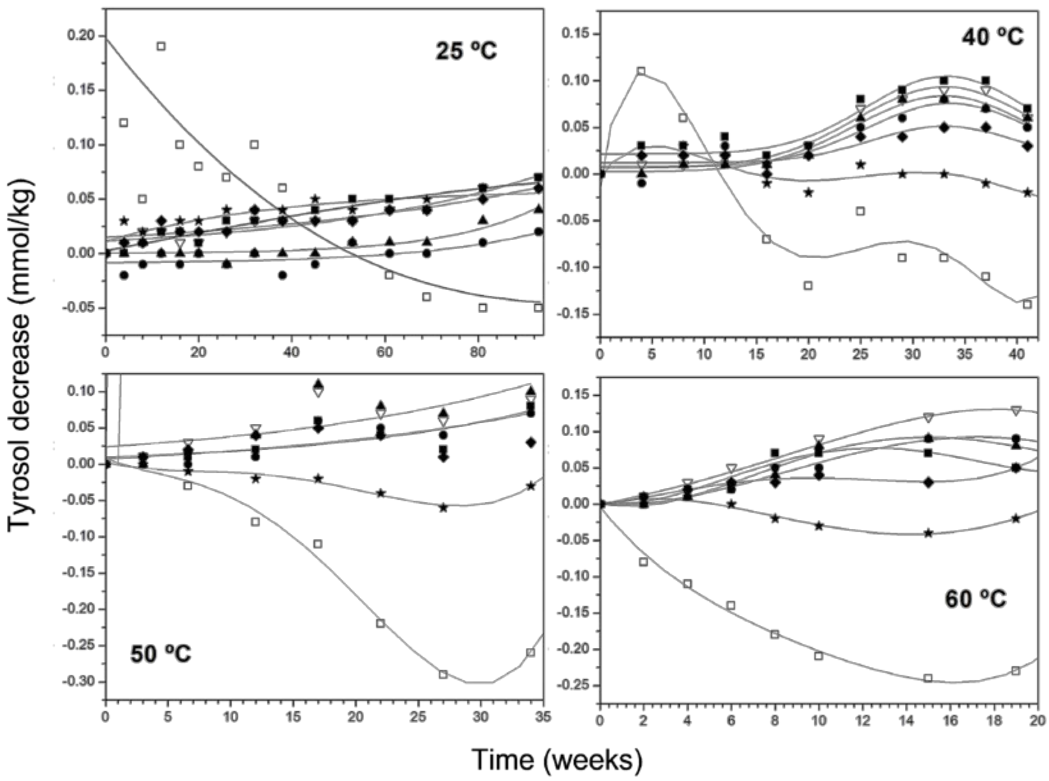

Figure S1. Decrease of tyrosol content in VOO samples at 25, 40, 50 and 60 ºC. Samples: ■, I; •, II; ▲, III; ∇, IV; ♦, V; □, VI; ★, VII.

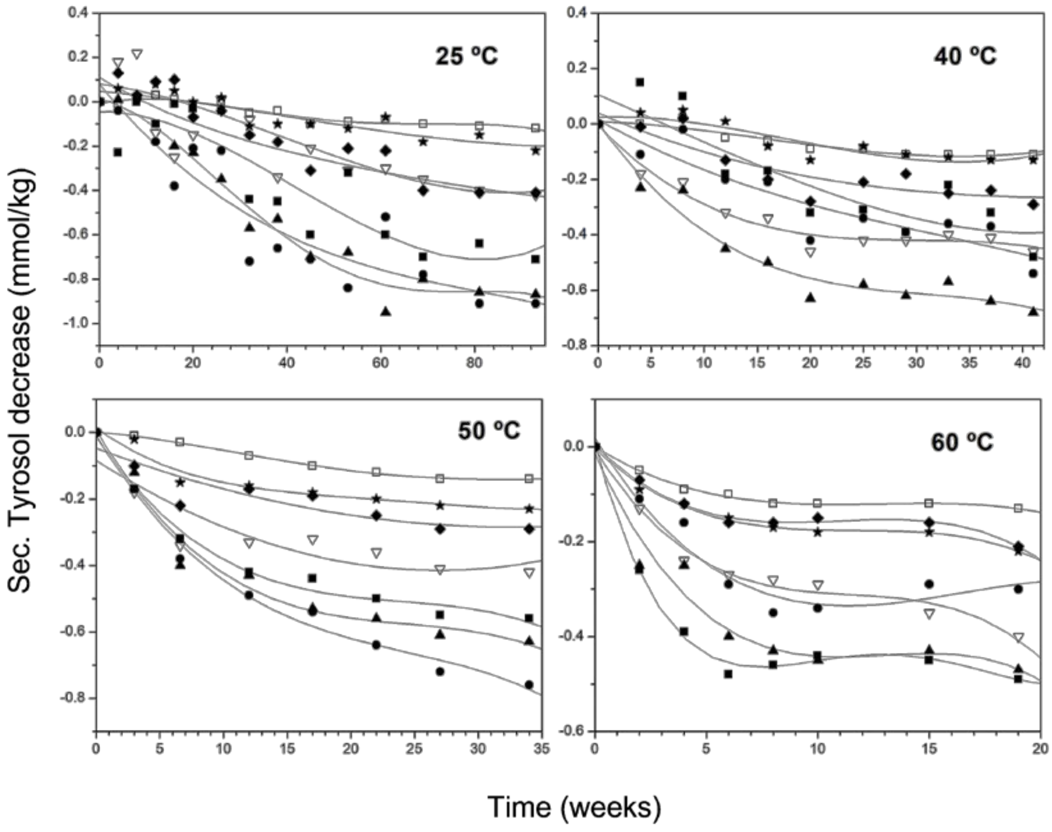

Figure S2. Decrease of tyrosol secoiridoid derivatives content in VOO samples at 25, 40, 50 and 60 ºC. Samples: ■, I; •, II; ▲, III; ∇, IV; ♦, V; □, VI; ★, VII.

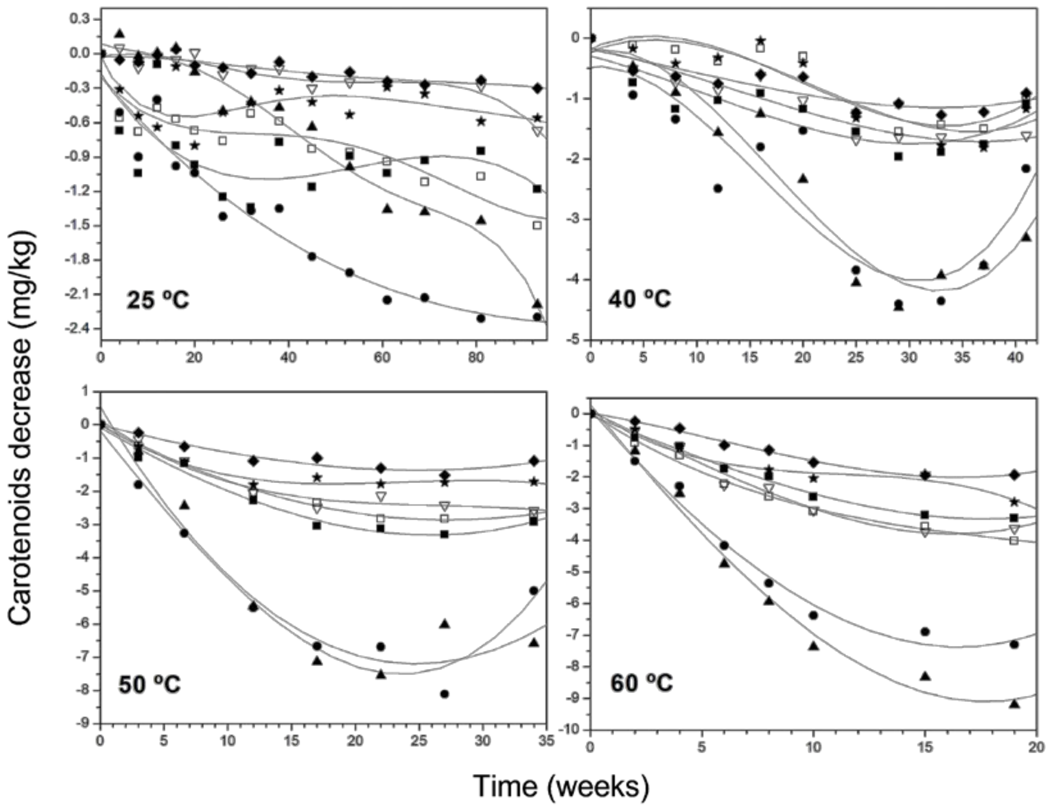

Figure S3. Decrease of carotenoid content in VOO samples at 25, 40, 50 and 60 ºC ■, I; •, II; ▲, III; ∇, IV; ♦, V; □, VI; , VII

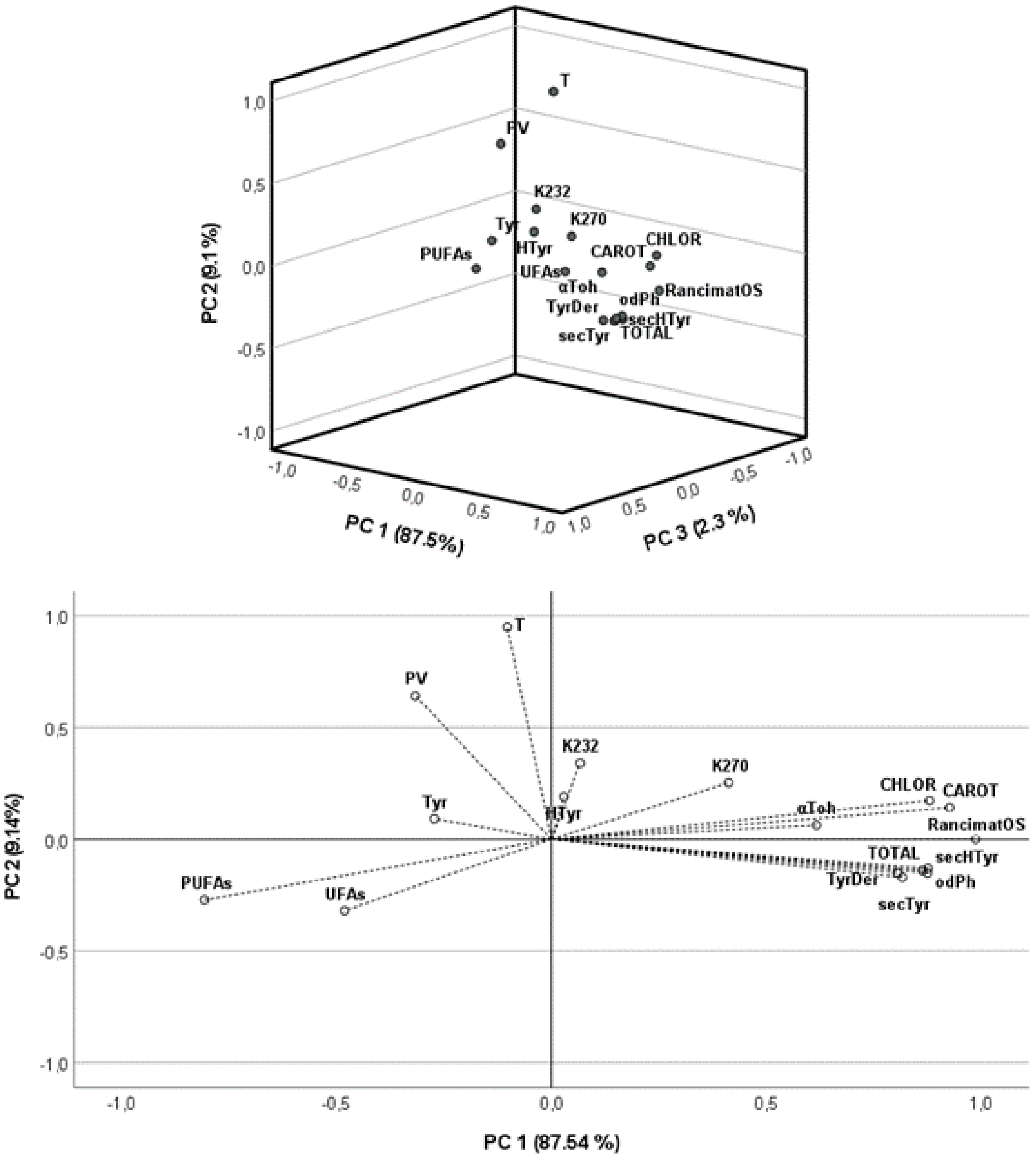

Figure S4. Loading plots of the principal component analysis (PCA) for variables related to the initial physical-chemical state of samples

Table S1. Kinetic parameters of complex phenols fitted to a pseudo first-order reaction: $Ln(C_t/C_0) = kt$, where C_0 is the initial concentration and C_t the residual concentration at time t.

		25 ºC			40 ºC			50 ºC			60 ºC		
		**k /10^{-2}**	**Ln C_0**	***R^2**	**k /10^{-2}**	**Ln C_0**	***R^2**	**k /10^{-2}**	**Ln C_0**	***R^2**	**k /10^{-2}**	**Ln C_0**	***R^2**
**Sec. Hydroxytyrosol**	**I**	-0,98 ± 0,08	0.666 ± 0.03	0.949	-4.23 ± 0.37	0.335 ± 0.08	0.930	-9.39 ± 0.19	0.273 ± 0.04	0.995	-17.23 ± 0.77	0.176 ± 0.09	0.979
	**II**	-1,04 ± 0,05	0.699 ± 0.04	0.925	-3.01 ± 0.18	0.289 ± 0.06	0.927	-8.43 ± 0.39	0.411 ± 0.03	0.997	-14.03 ± 0.62	0.225 ± 0.10	0.946
	**III**	-1.58 ± 0.14	0.799 ± 0.05	0.956	-8.22 ± 0.45	0.452 ± 0.14	0.932	-14.25 ± 0.13	0.293 ± 0.09	0.987	-23.30 ± 0.47	0.0005 ± 0.11	0.975
	**IV**	-1.47 ± 0.04	-0.183 ± 0.05	0.935	-8.69 ± 0.32	-0.492 ± 0.13	0.951	-12.35 ± 0.37	-0.807 ± 0.18	0.954	-33.10 ± 0.91	-0.673 ± 0.08	0.994
	**V**	-1.67 ± 0.12	-0.202 ± 0.05	0.939	-7.11 ± 0.51	-0.571 ± 0.15	0.894	-10.52 ± 0.45	-0.953 ± 0.15	0.951	-26.45 ± 0.10	-0.959 ± 0.06	0.996
	**VI**	-1.54 ± 0.05	-1.05 ± 0.04	0.953	-6.31 ± 0.17	-1.42 ± 0.10	0.956	-11.25 ± 0.24	-1.53 ± 0.08	0.987	-23.68 ± 0.49	-1.71 ± 0.06	0.994
	**VII**	-1.16 ± 0.07	-0.671 ± 0.04	0.941	-5.47 ± 0.20	-1.14 ± 0.11	0.923	-15.00 ± 0.62	-1.10 ± 0.08	0.991	-25.50 ± 0.35	-1.34 ± 0.07	0.995
**Sec. Tyrosol**	**I**	-0.86 ± 0.07	0.461 ± 0.06	0.739	-1.73 ± 0.16	0.016 ± 0.08	0.763	-2.43 ± 0.13	-0.052 ± 0.05	0.918	-10.35 ± 0.83	-0.008 ± 0.04	0.977
	**II**	-1.03 ± 0.10	0.459 ± 0.07	0.786	-1.53 ± 0.15	0.010 ± 0.06	0.821	-3.28 ± 0.18	0.095 ± 0.04	0.968	-5.94 ± 0.42	-0.076 ± 0.02	0.980
	**III**	-1.42 ± 0.04	0.271 ± 0.07	0.883	-3.08 ± 0.14	-0.323 ± 0.13	0.777	-5.04 ± 0.09	-0.327 ± 0.09	0.944	-9.35 ± 0.58	-0.471 ± 0.09	0.922
	**IV**	-0.94 ± 0.04	-0.166 ± 0.07	0.753	-2.15 ± 0.11	-0.658 ± 0.11	0.738	-3.13 ± 0.21	-0.781 ± 0.14	0.719	-13.00 ± 0.70	-0.716 ± 0.07	0.964
	**V**	-1.24 ± 0.13	-0.190 ± 0.07	0.818	-2.28 ± 0.02	-0.777 ± 0.11	0.749	-2.82 ± 0.33	-0.908 ± 0.10	0.794	-9.55 ± 0.44	-1.02 ± 0.01	0.999
	**VI**	-0.94 ± 0.07	-1.36 ± 0.04	0.889	-2.90 ± 0.18	-1.68 ± 0.08	0.907	-4.82 ± 0.24	-1.59 ± 0.04	0.987	-12.98 ± 0.60	-1.84 ± 0.06	0.976
	**VII**	-0.63 ± 0.09	-0.541 ± 0.04	0.817	-1.68 ± 0.33	-1.06 ± 0.10	0.675	-3.02 ± 0.22	-1.07 ± 0.08	0.873	-7.30 ± 0.60	-1.21 ± 0.06	0.936
**o-diphenols**	**I**	-0.87 ± 0.05	0.742 ± 0.03	0.941	-3.62 ± 0.29	0.457 ± 0.07	0.923	-8.20 ± 0.53	0.408 ± 0.07	0.987	-14.88 ± 0.57	0.307 ± 0.07	0.988
	**II**	-1.00 ± 0.12	0.739 ± 0.04	0.908	-2.51 ± 0.17	0.358 ± 0.06	0.910	-6.77 ± 0.42	0.454 ± 0.06	0.986	-12.28 ± 0.56	0.305 ± 0.08	0.951
	**III**	-1.46 ± 0.12	0.841 ± 0.05	0.942	-5.57 ± 0.34	0.427 ± 0.06	0.975	-11.98 ± 0.13	0.388 ± 0.08	0.986	-17.00 ± 1.50	0.062 ± 0.11	0.970
	**IV**	-1.29 ± 0.07	-0.163 ± 0.05	0.911	-3.89 ± 0.49	-0.728 ± 0.14	0.842	-11.48 ± 0.15	-0.672 ± 0.13	0.973	-31.98 ± 1.56	-0.498 ± 0.13	0.983
	**V**	-1.24 ± 0.09	-0.142 ± 0.05	0.916	-3.71 ± 0.33	-0.553 ± 0.07	0.948	-10.67 ± 0.63	-0.534 ± 0.10	0.984	-25.90 ± 0.78	-0.589 ± 0.07	0.994
	**VI**	-0.95 ± 0.04	-0.430 ± 0.02	0.962	-4.92 ± 0.18	-0.601 ± 0.07	0.957	-12.05 ± 0.58	-0.517 ± 0.07	0.994	-25.33 ± 0.25	-0.684 ± 0.10	0.989
	**VII**	-0.77 ± 0.05	-0.509 ± 0.03	0.897	-4.83 ± 0.18	-0.685 ± 0.11	0.903	-15.83 ± 0.57	-0.602 ± 0.17	0.966	-26.10 ± 1.15	-0.901 ± 0.07	0.994
**Total phenols**	**I**	-0.85 ± 0.01	1.35 ± 0.04	0.866	-1.89 ± 0.28	0.938 ± 0.07	0.840	-4.18 ± 0.11	0.924 ± 0.06	0.964	-10.98 ± 0.64	0.928 ± 0.04	0.980
	**II**	-0.97 ± 0.01	1.31 ± 0.05	0.845	-1.50 ± 0.25	0.879 ± 0.05	0.844	-4.29 ± 0.23	1.01 ± 0.05	0.974	-8.73 ± 0.44	0.870 ± 0.04	0.972
	**III**	-1.39 ± 0.13	1.28 ± 0.05	0.909	-2.81 ± 0.18	0.708 ± 0.13	0.772	-5.35 ± 0.44	0.695 ± 0.12	0.911	-14.75 ± 2.07	0.636 ± 0.11	0.933
	**IV**	-1.08 ± 0.10	0.563 ± 0.06	0.804	-2.27 ± 0.23	0.053 ± 0.09	0.789	-4.08 ± 0.09	-0.016 ± 0.13	0.825	-13.03 ± 0.74	0.010 ± 0.08	0.958
	**V**	-1.15 ± 0.12	0.567 ± 0.06	0.838	-2.41 ± 0.15	0.104 ± 0.06	0.896	-4.29 ± 0.14	0.026 ± 0.08	0.926	-11.53 ± 0.45	-0.098 ± 0.03	0.994
	**VI**	-0.66 ± 0.03	0.442 ± 0.03	0.852	-2.08 ± 0.12	0.209 ± 0.06	0.893	-4.55 ± 0.08	0.310 ± 0.02	0.995	-10.93 ± 0.13	0.172 ± 0.03	0.990
	**VII**	-0.59 ± 0.05	0.254 ± 0.04	0.738	-2.09 ± 0.08	-0.053 ± 0.07	0.849	-4.78 ± 0.14	-0.103 ± 0.08	0.940	-10.93 ± 0.62	-0.213 ± 0.04	0.985

k, reaction rate (weeks -1)

Ln C_0, obtained from the intercept for t=0

*$P<0.05$

(N = 4)

Table S2. Kinetic parameters of simple phenols and α-tocopherol fitted to a pseudo zero-order reaction: $C_t = C_0 + kt$

		25 ºC			40 ºC			50 ºC			60 ºC		
		***k* /10 $^{-3}$**	**C_0**	**$^*R^2$**	***k* /10 $^{-3}$**	**C_0**	**$^*R^2$**	***k* /10 $^{-3}$**	**C_0**	**$^*R^2$**	***k* /10 $^{-3}$**	**C_0**	**$^*R^2$**
HTYR	**I**	0.502 ± 0.12	0.170 ± 0.005	0.608	-1.47 ± 0.70	0.195 ± 0.01	0.425	-8.97 ± 0.72	0.238 ± 0.007	0.981	-12.57 ± 1.07	0.199 ± 0.006	0.972
	**II**	0.405 ± 0.09	0.099 ± 0.004	0.622	1.32 ± 0.27	0.103 ± 0.006	0.753	-2.73 ± 0.22	0.157 ± 0.003	0.968	-2.97 ± 0.44	0.127 ± 0.004	0.900
	**III**	0.379 ± 0.07	0.118 ± 0.003	0.775	-0.153 ± 0.26	0.140 ± 0.006	0.041	-4.16 ± 0.46	0.153 ± 0.005	0.965	-9.63 ± 0.60	0.171 ± 0.005	0.981
	**IV**	0.437 ± 0.06	0.035 ± 0.002	0.829	-0.479 ± 0.14	0.058 ± 0.003	0.589	-3.07 ± 0.54	0.069 ± 0.005	0.916	-5.39 ± 0.66	0.058 ± 0.004	0.944
	**V**	0.535 ± 0.08	0.078 ± 0.003	0.781	-1.93 ± 0.28	0.124 ± 0.005	0.887	-6.63 ± 0.63	0.146 ± 0.006	0.974	-10.61 ± 0.66	0.117 ± 0.004	0.985
	**VI**	-1.44 ± 0.14	0.315 ± 0.006	0.903	-6.76 ± 0.66	0.281 ± 0.01	0.946	-16.21 ± 1.08	0.308 ± 0.01	0.987	-28.06 ± 2.65	0.268 ± 0.016	0.966
	**VII**	0.413 ± 0.12	0.097 ± 0.005	0.484	-3.80 ± 0.81	0.168 ± 0.01	0.787	-8.51 ± 0.33	0.151 ± 0.003	0.996	-11.33 ± 1.15	0.110 ± 0.007	0.961
TYR	**I**	0.672 ± 0.06	0.1272 ± 0.003	0,907	2.69 ± 0.40	0.131 ± 0.009	0,849	1.80 ± 0.58	0.172 ± 0.01	0,621	5.51 ± 1.01	0.143 ± 0.008	0,857
	**II**	0.251 ± 0.07	0.090 ± 0.003	0,468	2.23 ± 0.31	0.076 ± 0.007	0,864	2.31 ± 0.48	0.112 ± 0.009	0,791	6.51 ± 0.71	0.088 ± 0.006	0,944
	**III**	0.3724± 0.07	0.079 ± 0.003	0,658	2.51 ± 0.58	0.078 ± 0.01	0,754	3.06 ± 0.75	0.119 ± 0.01	0,735	7.02 ± 0.82	0.093 ± 0.007	0,936
	**IV**	0.588 ± 0.04	0.043 ± 0.002	0,949	2.78 ± 0.33	0.051 ± 0.007	0,901	2.62 ± 0.65	0.080 ± 0.01	0,733	8.16 ± 0.49	0.057 ± 0.004	0,983
	**V**	0.514 ± 0.06	0.066 ± 0.003	0,869	1.41 ± 0.25	0.080 ± 0.005	0,802	0.674 ± 0.48	0.116 ± 0.009	0,244	2.31 ± 0.49	0.093 ± 0.005	0,786
	**VI**	-1.76 ± 0.43	0.646 ± 0.02	0,567	-8.46 ± 3.62	0.603 ± 0.04	0,577	-11.05 ± 1.28	0.634 ± 0.02	0,938	-20.04 ± 1.83	0.564 ± 0.01	0,968
	**VII**	0.446 ± 0.08	0.107 ± 0.004	0,704	-0.518 ± 0.38	0.165 ± 0.008	0,189	-2.04 ± 0.18	0.163 ± 0.003	0,965	-3.13 ± 0.64	0.144 ± 0.005	0,829
TOH	**I**	-1.44 ± 0.06	0.558 ± 0.008	0.936	-7.49 ± 0.62	0.512 ± 0.02	0.974	-25.20 ± 0.27	0.535 ± 0.04	0.973	-54.15 ± 0.37	0.554 ± 0.02	0.986
	**II**	-0.74 ± 0.04	0.445 ± 0.007	0.859	-3.80 ± 0.30	0.405 ± 0.01	0.895	-15.20 ± 0.22	0.440 ± 0.03	0.973	-38.05 ± 0.39	0.458 ± 0.02	0.970
	**III**	-1.25 ± 0.12	0.546 ± 0.01	0.887	-6.18 ± 0.39	0.493 ± 0.02	0.912	-25.80 ± 0.50	0.559 ± 0.03	0.985	-49.45 ± 0.47	0.566 ± 0.02	0.981
	**IV**	-0.99 ± 0.03	0.400 ± 0.01	0.815	-5.80 ± 0.15	0.362 ± 0.01	0.966	-20.83 ± 0.52	0.378 ± 0.003	1.000	-46.40 ± 0.18	0.410 ± 0.01	0.994
	**V**	-0.91 ± 0.03	0.466 ± 0.007	0.886	-5.24 ± 0.13	0.412 ± 0.01	0.949	-19.65 ± 1.39	0.431 ± 0.02	0.994	-46.98 ± 1.33	0.454 ± 0.02	0.982
	**VI**	-1.01 ± 0.14	0.335 ± 0.007	0.905	-3.47 ± 0.25	0.281 ± 0.006	0.97	-16.25 ± 0.53	0.311 ± 0.02	0.992	-34.58 ± 0.54	0.326 ± 0.01	0.984
	**VII**	-0.80 ± 0.04	0.359 ± 0.008	0.819	-3.63 ± 0.14	0.309 ± 0.008	0.949	-16.13 ± 2.11	0.336 ± 0.03	0.970	-36.60 ± 0.53	0.356 ± 0.009	0.995

k. reaction rate (mmol kg^{-1} weeks $^{-1}$)
Co. obtained from the intercept for t=0
*P<0.05
(*N* = 4)

Table S3. Components, loadings and scores of the PCA2

Component matrix [a] (loadings)		Components score matrix
	Component 1	Component 1
T	-0.917	-0.096
kPV	-0.952	-0.099
kK_{232}	-0.966	-0.101
kK_{270}	-0.967	-0.101
kTPh	0.969	0.101
kodPh	0.960	0.100
ksecHTyr	0.981	0.102
ksecTyr	0.944	0.098
$k\alpha$Toh	0.953	0.099
kUFAs	0.627	0.065
kPUFAs	0.981	0.102
Extraction method: Principal Components Analysis		Rotation method: Varimax.
[a]. 1 extracted component		Components score
